# Early relational origins of Theory of Mind: A two‐study replication

**DOI:** 10.1111/jcpp.14029

**Published:** 2024-07-18

**Authors:** Grazyna Kochanska, Lilly Bendel‐Stenzel, Danming An, Neevetha Sivagurunathan

**Affiliations:** ^1^ Department of Psychological and Brain Sciences The University of Iowa Iowa City IA USA; ^2^ Department of Psychology Lehigh University Bethlehem PA USA

**Keywords:** Theory of mind, parental mind‐mindedness, mutually responsive orientation

## Abstract

**Background:**

Research implies early relational factors – parental appropriate mind‐mindedness (MM) and mutually responsive orientation (MRO) – as antecedents of children's Theory of Mind (ToM), yet the longitudinal path is unclear. Furthermore, little is known about the process in father–child relationships. In two studies of community families in a Midwestern state in United States, we tested a path from parental appropriate MM in infancy to parent–child MRO in toddlerhood to children's ToM at preschool age in mother– and father–child relationships, using comparable observational measures at parallel ages.

**Methods:**

In Children and Parents Study (CAPS) of children born in 2017 and 2018, we collected data at 8 months (*N* = 200, 96 girls), 38 months, age 3 (*N* = 175, 86 girls), and 52 months, age 4.5 (*N* = 177, 86 girls). In Family Study (FS) of children born mostly in 2001, we collected data at 7 months (*N* = 102, 51 girls), 38 months, age 3 (*N* = 100, 50 girls), and 52 months, age 4.5 (*N* = 99, 49 girls). Parental MM (verbal comments aligned with the infant's psychological state) was observed in infancy, MRO (parent and child responsiveness to each other and shared positive affect) at age 3, and ToM (false belief tasks) at age 4.5.

**Results:**

The findings supported the proposed indirect effects of parents' MM on children's ToM, mediated by MRO, for fathers and children in both studies, and for mothers and children, in CAPS. In FS, mothers' MM predicted MRO and ToM, but there was no mediation.

**Conclusions:**

This investigation, testing a path from MM to MRO to ToM in both mother– and father–child relationships in two longitudinal studies, adds to the literature that has described relations among those constructs but rarely integrated those in one model.

Over the past 3 to 4 decades, research on children's Theory of Mind (ToM) has flourished in developmental psychology and psychopathology. ToM is a general concept encompassing various aspects of one's awareness and understanding of others' internal mental states – thoughts, beliefs, desires, emotions, or perspectives. Although early precursors are present at toddler age, ToM skills typically emerge between 4 and 5 years of age (Wellman, Cross, & Watson, 2001). It is a crucial social‐cognitive landmark, with broad implications for social functioning, peer relations, self‐regulation, school readiness, and sociomoral competence (Goffin, Kochanska, & Yoon, 2020; Hughes & Leekam, 2004; Imuta, Henry, Slaughter, Selcuk, & Ruffman, 2016; Slaughter, Imuta, Peterson, & Henry, 2015; Thompson, 2006, 2012). Deficits in ToM skills are associated with psychopathology, mostly autism, but also externalizing and internalizing problems (Brüne & Brüne‐Cohrs, 2006; Hughes & Ensor, 2006), and indeed, considered a transdiagnostic clinical marker (Cotter et al., 2018).

Research on predictors of ToM skills has grown rapidly (Hughes et al., 2005). Although early research had targeted mostly cognitive factors, such as language and executive functioning (Carlson & Moses, 2001; Devine & Hughes, 2014; Milligan, Astington, & Dack, 2007), interest in family factors, often traced to Dunn and colleagues' work (Dunn, Brown, Slomkowski, Tesla, & Youngblade, 1991), has been growing, including various aspects of the early parent–child relationship. That work has now produced multiple meta‐analytic and narrative reviews.

Two key sets of early relational characteristics have been most often associated with children's development of ToM. One set can be broadly described as parental mentalization. Growing literature has linked parental use of mental state talk, reflective functioning, and mind‐mindedness (MM) with children's social cognition generally, and ToM specifically (Aldrich, Chen, & Alfieri, 2021; Bernier, Lapolice‐Thériault, Matte‐Gagné, & Cyr, 2023; Colonnesi, Zeegers, Majdandzic, van Steensel, & Bögels, 2019; Devine & Hughes, 2018, 2019; Hughes, Devine, & Wang, 2018; Kirk et al., 2015; McMahon & Bernier, 2017; Slade, 2005).

The other set encompasses dyadic qualities of the parent–child relationship, such as security or positive mutuality. Secure attachment has been linked to children's better ToM skills (Fonagy & Target, 1997; Symons & Clark, 2000; Szpak & Bialecka‐Pikul, 2020; Thompson, 2006; Zeegers, Meins, Stams, Bogels, & Colonnesi, 2019). Early mutually responsive orientation (MRO; Kim & Kochanska, 2012; Kochanska, 1997, 2002; Kochanska & Murray, 2000) – a close, coordinated relationship, with the parent and child responsive to each other, and their interactions infused with shared positive affect – uniquely predicted children's ToM development 2–3 years later (Aubuchon et al., 2023). Positive mutuality, encompassing the parent's and the child's responsiveness to each other, was associated with the child's concurrent ToM (unless the household chaos was very high; McCormick, Chary, & Deater‐Deckard, 2022). Dyadic aspects of mother–child interaction at 7 months, especially a code integrating mutual responsiveness and affection, thus largely resembling MRO (‘clinical screener’), predicted child ToM at 50 months (Licata, Kristen, & Sodian, 2016).

Furthermore, research supports links between those two sets of predictors of ToM – parental mentalization and qualities of the parent–child early relationship. Parental mentalization, including appropriate MM, has been linked to aspects of a positive relationship, including secure attachment and parent–child mutual responsiveness and warmth (Bendel‐Stenzel, An, & Kochanska, 2024; Grienenberger, Kelly, & Slade, 2005; Kalland, Rutherford, & Pajulo, 2023; Katznelson, 2014; Koren‐Karie, Oppenheim, Dolev, Sher, & Etzan‐Carasso, 2002; Luyten, Mayes, Nijssens, & Fonagy, 2017; McMahon & Bernier, 2017; Meins, Fernyhough, Fradley, & Tuckey, 2001; Miller, Kim, Boldt, Goffin, & Kochanska, 2019; Slade, 2005; Zeegers, Colonnesi, Stams, & Meins, 2017). Interventions targeting parental mentalization have improved parent–child relational qualities (Camoirano, 2017; Slade, 2023; Stuhrmann, Göbel, Bindt, & Mudra, 2022; Suchman, DeCoste, Borelli, & McMahon, 2018).

We note that several meta‐analyses have revealed nuances in those relations and varying effect sizes. For example, Devine and Hughes (2018) concluded that both aspects of parental mentalization – mental state talk and appropriate MM – were modestly associated with children's ToM, but in a later study reported that whereas it was true for concurrent links, only mental state talk predicted future ToM (Devine & Hughes, 2019). Aldrich et al. (2021) reported *r* = .17 for MM – social cognition link (with social cognition encompassing ToM and several related skills). Effect sizes for parental mentalization – attachment links were reported as moderate (*r* = .30, Zeegers et al., 2017), but stronger for appropriate MM specifically. The attachment – ToM links ranged from modest (*r* = .19, Zeegers et al., 2019) to moderate (*r* = .30, Szpak & Bialecka‐Pikul, 2020). Some studies have failed to find the link (Ontai & Thompson, 2008). Of note, some studies cited in the meta‐analyses did not control for established correlates of ToM, such as children's language ability, which may account for the heterogeneous effect sizes. Nevertheless, after adjusting for the effect of language in the meta‐analyses, modest but robust associations between parental MM and parent–child attachment and children's ToM skills are still supported (*r* = .19 for MM and *r* = .10 for attachment; Devine & Hughes, 2018; Zeegers et al., 2019).

Thus, although the rapidly growing research supports links among all three sets of constructs – various forms of parental mentalization, quality of the early parent–child relationship, and children's ToM – the three literatures have been poorly integrated, in that the specific developmental paths elucidating those links have rarely been formally tested in a longitudinal design, even in studies that have included all three constructs.

Lundy (2013) examined whether mother– and father–child quality of interaction in a task mediated the effect of parental MM on children's ToM and supported the indirect effect for mothers and children. However, all measures were concurrent, the sample was small, and MM was assessed in an interview and not behaviorally. Laranjo, Bernier, Meins, and Carlson (2010) assessed mothers' MM at age 1, mother–child security at 16 months, and early ToM precursors at 26 months (and in a follow‐up study, also at age 4, Laranjo, Bernier, Meins, & Carlson, 2014). Both MM and security were positively associated with early and later ToM, but the authors did not test mediation. Licata et al. (2016) found that although mothers' MM at 7 months was associated with both concurrent positive mutuality and that at 50 months, only mutuality (at both times), but not MM, was associated with ToM at 50 months. Mediation through mutuality was not formally tested. Meins et al. (2002) examined mothers' MM in infancy, security of attachment at 12 months, and ToM at 45 and 48 months. They found that although appropriate MM was associated with security and with ToM, the latter two constructs were unrelated.

Consequently, the research on relations among parental MM, parent–child relationship, and children's ToM is inconclusive. McMahon and Bernier (2017), discussing parental MM – future child ToM links, emphasized that our understanding of the actual mechanisms involved is incomplete, and argued for prospective designs to elucidate those. To address this gap, we propose a straightforward mediational model: We expect that parental MM in infancy promotes parent–child MRO at toddler age, and MRO, in turn, promotes ToM skills at preschool age.

Furthermore, we address another large gap. Almost all extant studies have focused on mother–child relationships (e.g., Aldrich et al., 2021, was unable to include studies with fathers' MM; four available studies were combined as ‘mothers and fathers’). And yet, several recent studies and reviews indicate that fathers' MM may be uniquely important (Bendel‐Stenzel et al., 2024; Bernier et al., 2023; Colonnesi et al., 2019; Gagné, Bernier, & McMahon, 2018; Lundy, 2013; Mora et al., 2023), although not all studies have supported this (Goffin et al., 2020). Yet, to our knowledge, not a single study examined the entire proposed path from early MM to later parent–child MRO to ToM in both mother– and father–child relationships.

We report data from a large ongoing longitudinal study, Children and Parents Study (CAPS), and archival data from an earlier Family Study (FS), with behavioral, well‐established, parallel measures available at comparable ages. We assessed parental MM by coding the parents' comments to their infants in naturalistic interactions in infancy (Meins & Fernyhough, 2015). We observed parent–child MRO in lengthy interactions (Kochanska, 1997, 2002), targeting its three core components: The parent's and the child's responsiveness to each other and shared positive affect. Children's ToM was assessed with standard false belief tasks (Hughes et al., 2005; Wellman et al., 2001).

## Children and Parents Study (CAPS)

### Participants

A total of 200 two‐parent community families from a US Midwestern area (a college town, a small city, and rural areas and towns), with typically developing infants, biological children born in 2017 and 2018, volunteered for the study (demographic information in Table S1). Families were mostly White, but in 20% of families, one or both parents were not White. MM data were collected at 8 months (*N* = 200, 96 girls), MRO at 38 months, age 3 (*N* = 175, 86 girls), and ToM at 52 months, age 4.5 (*N* = 177, 86 girls). Additionally, we assessed covariates: MRO at 8 months and child receptive vocabulary at 4.5 years. Data were collected during mother– and father–child 2–3‐h sessions (at 8 months at home, otherwise in laboratory), parallel for both parents, conducted by female experimenters (Es), and video‐recorded. Attrition at ages 3 and 4.5 was due to the COVID‐19 pandemic; also, some families completed online measures but did not participate in person (refer to Table 1 for *N*s). Parents completed informed consents. The University of Iowa IRB approved the study (CAPS, 201701705).

**Table 1:** Children and Parents Study: descriptive data and correlations among all measures

	Mother MM, age 8 months	Father MM, age 8 months	Mother–child MRO, age 8 months^a^	Father–child MRO, age 8 months^a^	Mother–child MRO, age 3 years^b^	Father–child MRO, age 3 years^b^	Child ToM, age 4.5 years	Child PPVT age 4.5 years
Mother MM, age 8 months	–	.25***	.18*	.02	.25**	.11	.14^+^	.11
Father MM, age 8 months		–	.11	.12^+^	.21**	.30***	.18*	.08
Mother–child MRO, age 8 Months^a^			–	.27***	.16^+^	.18*	.19*	.04
Father–child MRO, age 8 months^a^				–	−.07	.09	−.01	.02
Mother–Child MRO, age 3^b^					–	.30***	.30***	.19*
Father–child MRO, age 3^b^						–	.34***	.10
Child ToM, age 4.5							–	.48***
*M*	10.58	8.80	0.00	0.00	0.00	0.00	2.19	0.00
*SD*	7.12	6.80	1.00	1.00	0.81	0.83	2.14	1.00
Range	0–40	0–44	−3.36 to 2.07	−2.66 to 2.74	−2.39 to 1.41	−2.38 to 1.55	0.00 to 8.00	−4.28 to 2.25
*N*	200	200	200	200	157	149	157	152

^+^ *p* < .10; *p < .05; **p < .01; ***p < .001. MM, Mind‐Mindedness; ToM, theory of mind.

^a^ Shared positive affect (standardized).

^b^ A composite of the three standardized scores: the parent's and the child's responsiveness to each other and shared positive affect.

### Mothers' and fathers' appropriate MM, age 8 months

Parents' appropriate MM comments were coded during 10‐min snack context, for mother– and father–child dyads (see Bendel‐Stenzel et al., 2024). Coding followed Meins and Fernyhough (2015). First, transcribers wrote down verbatim each parental comment to the child. Second, while watching the videos, coders coded each comment as *MM* (references to the infant's desires, cognitions, emotions, and talking on the infant's behalf) or *not MM* (the latter were not considered further). Each MM comment was then coded as *appropriate* if the coder agreed with the parent's reading of the infant's internal state, the comment linked the infant's current activity with similar events in the past or future, or the comment served to clarify how to proceed after a lull in the interaction. The remaining MM comments were coded as *nonattuned* (very rare and not considered further, a common approach, e.g., Regueiro, Matte‐Gagné, & Bernier, 2022). Reliability, kappas, ranged from .96 to .99 for *MM* versus *not MM* comments, and .69 to .95 for *appropriate* versus *nonattuned* MM comments. Although some studies use proportional scores, results are often the same for frequency scores (Laranjo et al., 2014; McMahon & Bernier, 2017; Meins, Centifanti, Fernyhough, & Fishburn, 2013), and the latter tend to predict child outcomes better (Aldrich et al., 2021). Thus, for each parent, we tallied all appropriate MM comments and used those scores, consistent with past work (An & Kochanska, 2022; Bernier et al., 2023; Bernier, McMahon, & Perrier, 2017; Goffin et al., 2020; Miller et al., 2019); mothers produced more of those than fathers, *t*(199) = 2.95, *p* = .004. There were no differences due to child gender.

### Mother–child and father‐child MRO, age 3 years

#### The parent's responsiveness to the child

Parental responsiveness was coded during scripted, naturalistic contexts (total of 25 min for each parent–child dyad): introduction to the room (5 min), waiting for snack (5 min), snack (5 min), parent busy (5 min), and play (5 min). The parent received one overall rating for each context (e.g. snack, play), from 1 (*highly unresponsive*) to 7 (*highly responsive*), incorporated the classic dimensions (Ainsworth, Bell, & Stayton, 1971): sensitivity‐insensitivity, cooperation‐interference, and acceptance‐rejection.

Reliability, weighted kappas, were .87 to .92. The scores were averaged into an overall responsiveness score: mothers, *M* = 4.99, *SD* = 0.54, fathers, *M* = 4.70, *SD* = 0.68. The difference was significant, *t*(148) = 5.05, *p* < .001. Girls and boys did not differ in terms of responsiveness received from either parent.

#### The child's responsiveness to the parent

Child responsiveness was coded in three of the above contexts (introduction to the room, waiting for snack, and play; coded for each 1‐min segment) and in two elicited imitation contexts, when the parent demonstrated play scripts and asked the child to imitate (the child received one score for each part, demonstration, and imitation). The codes ranged from 1 (*not responsive*) to 5 (*highly responsive*) and were added for each context. The rating incorporated the child's sensitivity (detection, interpretation, and prompt, appropriate, and contingent response to the parent's cues or overtures, etc.), acceptance (warmth, enjoyment, affection, resentment toward the parent), and cooperation (respect for the parent, acknowledging his/her attempts). Generally, high scores denoted instances when the child's behavior was likely to please the parent. Reliability, weighted kappas, were .62 to .76. The scores were then averaged across the contexts into an overall responsiveness score for the child (with each parent): children with mothers, *M* = 14.02, *SD* = 2.06, and children with fathers, *M* = 13.40, *SD* = 1.93, a significant difference, *t*(148) = 3.99, *p* < .001. Girls were more responsive to mothers than boys, *M* = 14.46, *SD* = 1.93; and *M* = 13.59, *SD* = 2.11, *t*(155) = 2.71, *p* = .008, but there was no gender difference with fathers.

#### Parent–child shared positive affect

Parent and child affect was coded in the same contexts as parental responsiveness. For each 30‐s segment, coders coded expressions of positive and negative affect as 0 (*not present*), 1 (*neutral positive or neutral negative mood*; not a ‘full‐blown’ positive emotion, but upbeat, pleasant, engaged mood or fatigue, subtle discomfort, disengagement, negatively tinged mood), 2 (*discrete positive or negative emotion*; ‘full‐blown’ expression of affection or joy, distress, cry, anger, etc.), or 3 (*a strong positive or negative emotion*; intense or lasting more than 15 s). Reliability, weighted kappas, ranged from .70 to .87.

All segments in which both the parent and the child displayed positive affect or neutral positive mood and neither displayed negative affect or neutral negative mood were tallied for each context. We then computed the average of those tallies to create a score of shared positive affect for each dyad: mother–child dyads, *M* = 7.69, *SD* = 1.30; father–child dyads, *M* = 7.11, *SD* = 1.79, a significant difference, *t*(148) = 3.70, *p* < .001. Girls and boys had comparable scores, with either parent.

#### Parent–child MRO

The three scores correlated, for mothers, *r*'s .36–.56, *p*s < .001, and fathers, .44–.66, *p*s < .001, and were standardized and averaged into a composite of MRO for each dyad (see also Appendix S3). There were no differences due to children's gender.

### Children's ToM, age 4.5 years

#### False belief tasks

E presented the child with four well‐established tasks (Hughes et al., 2005): unexpected contents (Band‐Aid Box story), unexpected location (Andy's Apple and New Toy stories), and belief‐desire (Juice story). The tasks employed pictures, puppets, and props (e.g., a Band‐Aid box), as appropriate. Coding followed Hughes et al. (2005, pp. 369–370). Coders assigned scores of either 0 (incorrect answer) or 1 (correct answer) to each critical test question asked per story. To earn a point, the child had to responded correctly to both the critical test question (false belief question) and the control questions (e.g. ‘what is in the box really?’, ‘where is the toy really?’). Children did not earn any points if they did not first respond correctly to the control question(s). Reliability, kappas, ranged from .87 to 1.00. The total ToM score was the sum of scores across tasks. There was no difference due to child gender.

### Covariates

#### Parent–child MRO, age 8 months

We could not create a score of MRO fully parallel to age 3, because child responsiveness had not been coded in infancy. However, parent–child shared positive affect had been coded analogously to age 3, for mother– and father–child relationships, *M* = 9.07, *SD* = 2.63, and *M* = 7.52, *SD* = 2.55, respectively. It was used as a robust indicator of the dyadic quality of the early relationship; standardized, and covaried to control for the continuity of MRO.

#### Children's receptive language skills, age 4.5

We administered Peabody Picture Vocabulary Test (PPVT‐5; Dunn, 2019). The standardized scores were covaried as a control for ToM. All descriptive data are in Table 1.

### Results

#### Preliminary analyses

Families that returned at age 4.5 did not differ significantly on any construct from those that did not return. The intercorrelations are in Table 1. In both mother– and father–child relationships, appropriate MM comments at 8 months were associated with concurrent MRO (marginally for fathers) and with MRO at age 3, and with ToM at age 4.5 (marginally for mothers). MRO at age 3 (for mothers and children, also at 8 months) was associated with ToM. Both appropriate MM and MRO (at 8 months and at age 3) correlated across the two relationships. Children's PPVT scores correlated with ToM and with mother–child MRO at age 3.

#### Main analyses: indirect effects of MM on ToM, mediated by MRO

As noted, there was attrition at ages 3 and 4.5. Because PROCESS (Hayes, 2022) uses listwise deletion for missing data, we utilized the Mplus code provided by Stride, Gardner, Catley, and Thomas (2015). By converting the original SPSS PROCESS macro syntax into the Mplus program, this method allows for the use of the full information maximum likelihood (FIML) treatment for missing data within the framework of PROCESS (95% confidence intervals (CIs), of indirect effects were estimated using bias‐corrected bootstrapping with 10,000 resamples). We modeled parental appropriate MM at 8 months as the predictor, parent–child MRO at age 3 as the mediator, and ToM at age 4.5 as the outcome variable. The effect of child PPVT score on ToM was covaried. Furthermore, the effect of parent–child MRO at 8 months on MRO at age 3 was covaried, to test whether appropriate MM was associated with a *change* in MRO at age 3, controlling for continuity of MRO.

**Figure 1:**
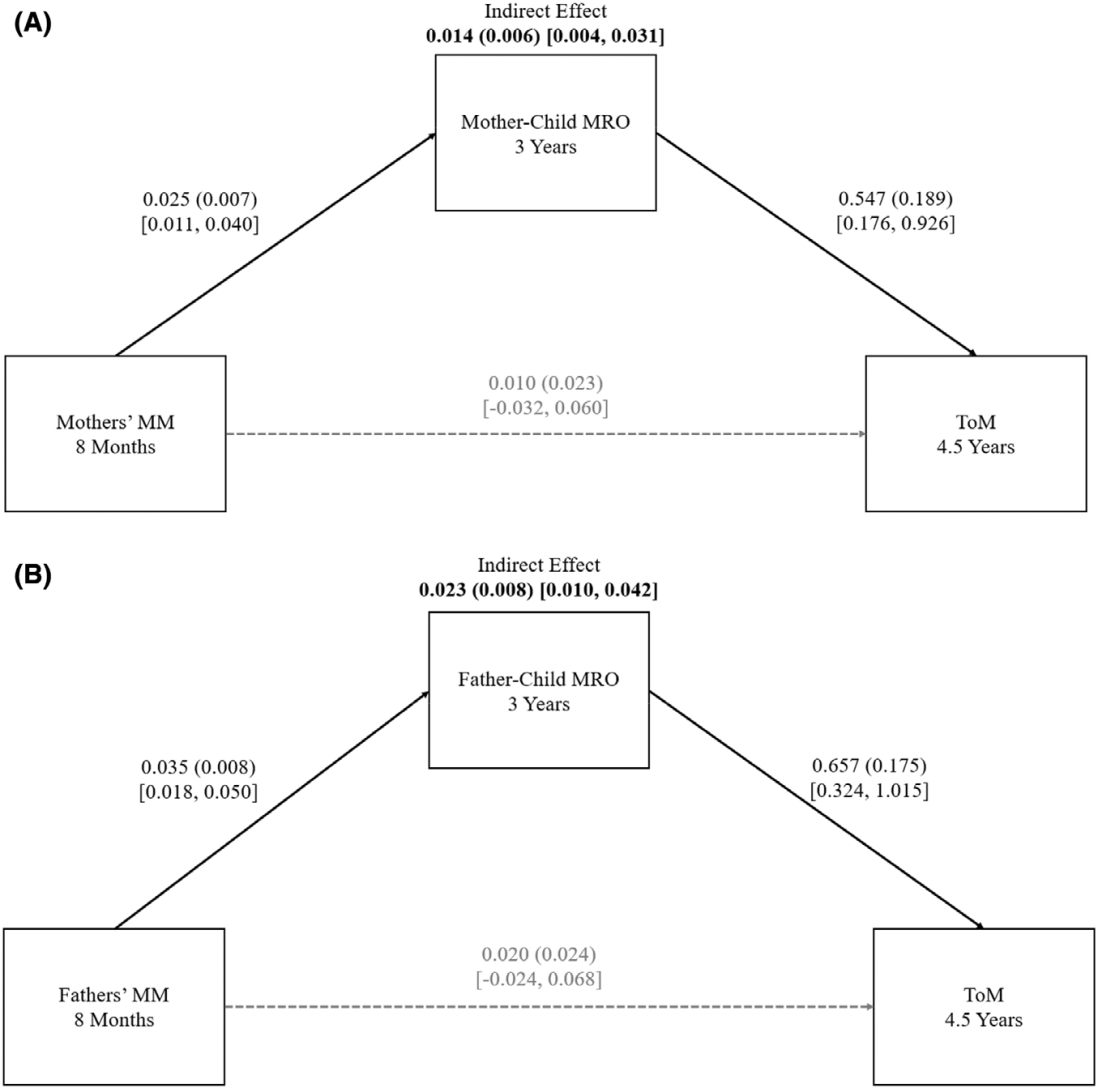
Children and Parents Study. Panel A: Mother–child dyads. Panel B: Father–child dyads. Mediation model: From the parent's MM to parent–child MRO to child ToM. Reported unstandardized coefficients, standard errors (in parentheses), and bootstrap 95% CIs [in brackets]. Solid lines represent significant effects; dashed lines represent non‐significant effects. Significant indirect effects are bolded. MM, appropriate mind‐mindedness; MRO, mutually responsive orientation; ToM, theory of mind. Child PPVT scores at 4.5 years and MRO at 8 months were covaried but are not depicted

The findings are in Figure 1 (Panel A, mother–child dyads, and Panel B, father–child dyads). The proposed mediational model was supported for both mother– and father–child dyads: The indirect effect of the parent's MM on ToM through MRO was present in both.

## Family Study (FS)

### Method

#### Participants and design

A total of 102 two‐parent, community families with typically developing infants, biological children born mostly in 2001, from the same area as in CAPS, volunteered for the study (demographic information in Table S2). Families were mostly White, but as in CAPS, in 20% of families, one or both parents were not White. Data on MM were collected at 7 months (*N* = 102, 51 girls), on MRO at 38 months, age 3 (*N* = 100, 50 girls), and on ToM at 52 months, age 4.5 (*N* = 99, 49 girls). Additionally, we assessed covariates: MRO at 7 months and parent‐reported child verbal skills at 15 months (*N* = 101, 50 girls). All data were collected during mother– and father–child 2–4‐h sessions, parallel for both parents, conducted by female Es and video‐recorded (at 7 months, at home; at age 3, at home and laboratory, and at age 4.5, in laboratory). Parents completed informed consents. The study was approved by University of Iowa IRB (Developmental Pathways to Antisocial Behavior: A Translational Research Program, 200107049).

#### Mothers' and fathers' appropriate MM, age 7 months

Parents' appropriate MM comments were coded during snack (7 min for the child with each parent, see Miller et al., 2019), following Meins and Fernyhough (2015). The approach was the same as in CAPS. Reliability, kappas, for the judgment *MM* versus *not MM*, ranged from .96 to .99, and for the judgment *appropriate* versus *nonattuned* they ranged from .69 to .95. Mothers made more appropriate MM comments than fathers, *t*(100) = 3.26, *p* = .002. There was no difference due to child gender.

#### Mother–child and father–child MRO, age 3 years

##### The parent's responsiveness to the child

Parental responsiveness was coded analogously to CAPS, for 77 min for each parent–child dyad; at home, play (5 min), toy cleanup (10 min), making cupcakes (20 min), and snack (15 min), and in laboratory, introduction to the room (5 min), snack (10 min), play (5 min), toy cleanup (5 min), and opening a gift (2 min). The parent received one overall rating for each context, from 1 (*highly unresponsive*) to 7 (*highly responsive*). Reliability, weighted kappas, ranged from .70 to .75. The scores were averaged across the contexts into an overall responsiveness score (home and laboratory correlated .59 and .57, *p*s < .001, for mothers and fathers). The mothers' scores were higher than fathers, *M* = 4.91, *SD* = 0.63, and *M* = 4.60, *SD* = 0.79, respectively, *t*(97) = 3.50, *p* < .001. There was no difference due to child gender.

##### The child's responsiveness to the parent

The same contexts were used to code the child's responsiveness to the parent. The approach was slightly different than in CAPS, although the definition of child responsiveness was the same (FS was chronologically first, and some coding systems were adapted in CAPS), and incorporated child sensitivity, acceptance, and cooperation, and behavior likely to please the parent. The child received one score for each context, from 1 (*highly unresponsive*) to 7 (*highly responsive*). Reliability, weighted kappa, was .76. The scores were averaged across the contexts into an overall responsiveness score for the child (with each parent; home and laboratory correlated .52 and .64, *p*s < .001, for mothers and fathers); children with mothers, *M* = 5.13, *SD* = 0.53, and children with fathers, *M* = 5.08, *SD* = 0.62, not a significant difference. There was no difference due to child gender.

##### Parent–child shared positive affect

The same contexts were coded for the parent's and child's affect, for each 30‐s segment. The logistics were slightly different than in CAPS, resulting in different metrics, but the codes were defined analogous to CAPS: *discrete positive affect*, *neutral positive mood*, *discrete negative affect*, and *neutral negative mood*. Intense discrete affects were marked. Reliability, kappas, were .86 for parents, .84 to .88 for children.

All segments in which both the parent and the child displayed discrete positive affect or neutral positive mood and neither displayed discrete negative affect or neutral negative mood were tallied, and those tallies were divided by the number of coded segments to create a score of shared positive affect for each dyad, separately for home and laboratory; those correlated .38 and .46, *p*s < .001, for mothers and fathers, and were averaged into final shared positive affect scores: mother–child dyads, *M* = 0.85, *SD* = 0.13, and father–child dyads, *M* = 0.80, *SD* = 0.14, a significant difference, *t*(97) = 2.69, *p* = .008. There was no difference due to child gender.

##### Parent–child MRO

The three scores correlated, for mothers, *r*'s .36–.60, *p*s < .001, and for fathers, .52–.77, *p*s < .001, and were standardized and then averaged into a composite of MRO for each dyad. There was no difference due to child gender.

#### Children's ToM, age 4.5

##### False belief tasks

E presented the child with the same four tasks as in CAPS. The minor differences involved Chocolate replacing New Toy, and Coke replacing Juice. Coding was parallel to CAPS (Hughes et al., 2005). Reliability, kappas, were 1.00, for all stories. The total ToM score was the sum of scores across tasks. There was no difference due to child gender.

#### Covariates

##### Parent–child MRO, age 7 months

To create an MRO score at 7 months parallel to CAPS, we again used shared positive affect, for mother– and father–child relationships, coded in infancy (tallies divided by the number of coded segments), *M* = 0.69, *SD* = 0.14, and *M* = 0.62, *SD* = 0.16, respectively. It was standardized and used as the best indicator of the dyadic quality of the early relationship and covaried to control for continuity of MRO.

##### Children's verbal skills, age 15 months

Because we did not have PPVT data, as a proxy for language development we drew from parents' reports of the child's development of self (Stipek, Gralinski, & Kopp, 1990). We selected 10 items that described verbal behavior (e.g., ‘uses words such as I, me, you, mine’; ‘uses descriptive terms, such as sticky, dirty, broken’), reported as 0 (*definitely has not shown*), 1 (*sort of*), or 2 (*definitely has shown*); *M* = .28, *SD* = .13, averaged across the two parents, and standardized. All descriptive data are in Table 2.

**Table 2:** Family Study: descriptive data and correlations among all measures

	Mother MM, age 7 months	Father MM, age 7 months	Mother–child MRO, age 7 months^a^	Father–child MRO, age 7 months^a^	Mother–child MRO, age 3 years^b^	Father–child MRO, age 3 years^b^	Child ToM, age 4.5 years	Verbal ability, age 15 months
Mother MM, Age 7 months	–	.26**	.22*	.36***	.29**	.20+	.31**	.08
Father MM, age 7 months		–	.09	.19+	.06	.22*	.24*	−.02
Mother–child MRO, age 7 months			–	.33***	.30**	.15	.16	−.00
Father–child MRO, age 7 months				–	.18+	.29**	.15	−.08
Mother–child MRO, age 3 years^a^					–	.41***	.22*	−.08
Father–child MRO, age 3 years^a^						–	.28**	−.05
Child ToM, age 4.5 years							–	.11
*M*	8.13	6.01	0.00	0.00	0.00	0.00	3.00	0.00
*SD*	5.09	5.61	1.00	1.00	0.80	0.86	2.64	1.00
Range	0–22	0–38	−2.48 to 2.02	−2.44 to 2.29	−2.61 to 1.36	−3.08 to 1.67	0.00 to 8.00	−1.69 to 4.69
*N*	101	101	102	102	99	99	98	101

^+^ *p* < .10; *p < .05; **p < .01; ***p < .001; MM, mind‐mindedness; ToM, Theory of Mind.

^a^ Shared positive affect (standardized).

^b^ A composite of the three standardized scores: the parent's and the child's responsiveness to each other and shared positive affect.

### Results

#### Preliminary analyses

The intercorrelations are in Table 2. In both mother– and father–child relationships, appropriate MM comments at 7 months were significantly associated with MRO at age 3 and at 7 months (marginally for fathers); and MM and MRO at age 3 were associated with ToM. Appropriate MM and MRO at 7 months and at age 3 correlated across the two relationships.

#### Main analyses: indirect effects of MM on ToM, mediated by MRO

Given the minimal attrition, we decided to proceed with listwise deletion. We tested our hypothesized mediation model using the PROCESS macro in SPSS 29 (10,000 bootstraps, 95% CIs; Hayes, 2022). We modeled parental appropriate MM at age 7 months as the predictor, parent–child MRO at age 3 as the mediator, and ToM at age 4.5 as the outcome variable. Again, we controlled for parent‐reported child verbal skills and parent–child MRO at 7 months.

**Figure 2:**
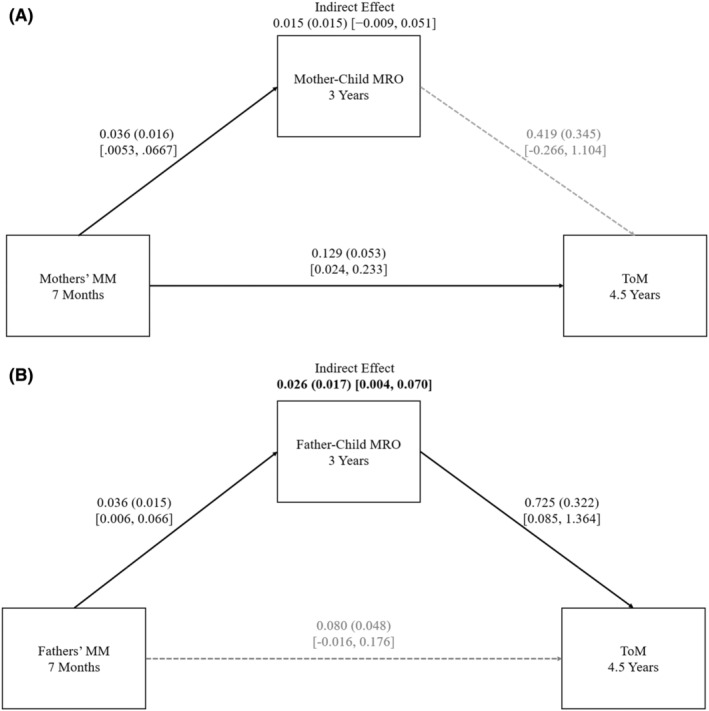
Family Study. Panel A: Mother–child dyads. Panel B: Father–child dyads. Mediation model: From the parent's MM to parent–child MRO to the child's ToM. Reported unstandardized coefficients, standard errors (in parentheses), and bootstrap 95% CIs [in brackets]. Solid lines represent significant effects; dashed lines represent non‐significant effects. Significant indirect effects are bolded. MM, appropriate mind‐mindedness; MRO, mutually responsive orientation; ToM, theory of mind. Child verbal skills at 15 months and MRO at 7 months were covaried but are not depicted

The findings are in Figure 2 (Panel A, mother–child, and Panel B, father–child dyads). In mother–child dyads, the mediational model was not supported. Mothers' MM was associated with mother–child MRO at age 3 and children's ToM, but MRO was unrelated to ToM. There was no indirect effect of mothers' MM on children's ToM through MRO.

For father–child dyads, the mediational model was supported. The expected indirect effect of fathers' MM on ToM through father–child MRO was present.

## General discussion

Direct associations between parental MM and ToM are well known; that research has matured to the point of producing several comprehensive reviews and meta‐analyses (Aldrich et al., 2021; Devine & Hughes, 2018; McMahon & Bernier, 2017). The reviews highlight nuances of those associations regarding types and measures of parental mentalization and of ToM. They also specifically articulate the need to understand *mechanisms* that account for such associations (McMahon & Bernier, 2017). Although several have been proposed in the cognitive tradition, as socialization researchers, we find the possibility that relational processes may link early parental appropriate references to the infant's mental states and the child's understanding of others' minds at preschool age perhaps most intriguing.

Indeed, links between parental MM (and other forms of mentalization) and parent–child attachment security have been broadly established (McMahon & Bernier, 2017; Miller et al., 2019; Slade, 2005; Zeegers et al., 2017), and they likely apply to parent–child MRO. Highly mentalizing parents may be more apt at reading and responding appropriately to infants' signals and creating positive mutual affective ambience. In turn, children may willingly appreciate the parents' perspective, further facilitating MRO and child self‐regulation, advancing children's social cognition broadly (Aldrich et al., 2021; Bendel‐Stenzel et al., 2024; Szpak & Bialecka‐Pikul, 2020; Zeegers et al., 2019). Highly mentalizing parents also provide the child with a rich linguistic input, effectively scaffolding their emerging social cognition. Most likely, early relational and cognitive influences work in concert and complement each other as catalysts helping young children understand their social world.

The extant literature broadly supports relations among parents' appropriate MM when interacting with their infants, the quality of the relationships they establish with their children, and the children's ToM development. To our knowledge, however, no study has explicitly investigated, in a longitudinal design, specific developmental pathways linking those constructs, in both mother– and father–child relationships, despite growing calls for more research that elucidates developmental processes in both relationships, including sequelae of parental MM (Aldrich et al., 2021; McMahon & Bernier, 2017; Volling & Cabrera, 2019).

In two longitudinal studies, with largely parallel measures, we tested a straightforward model, with parental appropriate MM in infancy promoting the child's ToM at preschool age indirectly, through MRO, a positive, mutually responsive relationship formed at toddler age, a construct rooted in theories of attachment, reciprocity (Maccoby & Martin, 1983), and communal orientation (Clark & Mills, 2012).

We largely supported that model. Although the univariate correlations were similar across FS and CAPS, and the indirect effects of early appropriate MM on future ToM via MRO were present for fathers and children in both studies, the indirect effect for mothers and children was supported only in CAPS. Perhaps with a larger sample, mediation for mothers and children would also be detected in FS. But the clear replication of the indirect effect for fathers and children highlights a robust, unique, yet poorly understood, role of fathers' MM in the origins of a developmental cascade promoting children's social cognition. These findings dovetail with growing – although still scarce – research (Bernier et al., 2023; Colonnesi et al., 2019; Gagné et al., 2018; Laflamme, Matte‐Gagne, & Baribeau‐Lambert, 2022). Bernier et al. (2023) suggested that fathers may express mind‐mindedness in particularly stimulating ways, accounting for their unique impact; however, this hypothesis is tentative, given that we did not examine nuanced differentiations within specific verbal comments in parents' appropriate MM.

This work has strengths and weaknesses. The former includes robust behavioral data, largely parallel in both studies. Furthermore, in CAPS, the model was supported above and beyond the impact of children's receptive vocabulary on ToM. In FS, a chronologically much earlier study, we did not assess children's vocabulary specifically, and thus we used a proxy measure derived from parental reports of child verbal behavior. This is a significant limitation and a reason for caution.

The longitudinal design from infancy to preschool age, allowing for testing the indirect effect of parental MM in infancy on ToM via the relationship quality at toddler age, was a strength in both studies. However, our ability to draw inferences about causality is limited. In both studies, we controlled for the effect of the parent–child relationship assessed in infancy on MRO at age 3. Therefore, we can claim that parental appropriate MM was associated with a *change* in MRO at age 3, above and beyond simple developmental continuity. We did not, however, have earlier measures of ToM, and therefore cannot claim with certainty that MRO was linked to change in ToM. It is entirely possible that child ToM may drive MRO, such that parents develop better relationships with children who are more skilled in understanding others' perspectives (peers', teachers', or parents') than with children who are less skilled in that regard. This is another significant limitation.

The children were typically developing and parent–child interactions in those community families were overall positive. Future research would benefit from including families with developmental risks, such as dysfunctional parent–child relationships (abuse, neglect), or children with elevated risk for psychopathology, including autism. Racial and ethnic diversity was limited, although in both studies, 20% of families (total *N* = 60) were not ‘White alone’.

Despite the limitations, the findings contribute to the growing evidence of the importance of early relational factors in children's emerging ToM, a key social‐cognitive competence. They also have translational implications by emphasizing the long‐term impact of parental appropriate mind‐related comments during interactions with infants, thus informing future early parenting intervention and prevention programs.

**Key points**

- Children's ToM skills – the awareness of and understanding others' mental states – constitute a key social‐cognitive competence. Individual differences in ToM are associated with multiple aspects of adaptive development and with psychopathology.
- Studying predictors of those differences, researchers have examined early relational qualities, including parental appropriate MM and parent–child MRO, with both seen as promoting ToM. Yet, the specific developmental path, in both mother and father relationships, has rarely been formally tested.
- In two longitudinal studies, we tested a straightforward mediational model that posited an indirect effect of parental appropriate MM in infancy on children's ToM at preschool age, mediated by parent–child MRO at toddler age, in both mother and father relationships.
- Both studies supported the model for fathers and children; one study supported it for mothers and children.
- The results inform basic research on ToM and parenting programs by elucidating the significance of parental appropriate MM in infancy, with long‐term cascading effects for social cognition.

## Supporting information


**Table S1.** CAPS: Demographic characteristics of the recruited sample at entry (*N* = 200).
**Table S2.** FS: Demographic characteristics of the recruited sample at entry (*N* = 102).
**Appendix S3.** The construction of MRO measure

## Data Availability

Although we will gladly share the coding system or the syntax used in the analyses, we are unable to share data for individual families. Our consent forms the parents signed clearly preclude any sharing of individual data, even if deidentified. The parents have consented to the data being shared in the aggregate form only, and we are ethically and legally bound to follow this agreement.
