# Supplementary material for: Early relational origins of Theory of Mind: A two‐study replication
Source: J Child Psychol Psychiatry. 2024 Jul 18;66(1):41–52. doi: 10.1111/jcpp.14029 (PMC11652414; doi:10.1111/jcpp.14029)
Supplement: Supplementary file 1 — Table S1. CAPS: Demographic characteristics of the recruited sample at entry (N = 200). Table S2. FS: Demographic characteristics of the recruited sample at entry (N = 102). Appendix S3. The construction of MRO measure [file JCPP-66-41-s001.zip › Appendix_S3_MRO_construction_June_17.docx]

# Early Relational Origins of Theory of Mind: A Two-Study Replication

# Appendix S3

*The construction of MRO measure*

We should note that in the current article, we created the measure of MRO by aggregating across its three separately coded key components – the parent’s and the child’s responsiveness to each other and shared positive affect (Kochanska & Murray, 2000) – rather than using a simplified dyadic code more typically deployed (Kim & Kochanska, 2012). This was because the dyadic coding was not yet available in CAPS, and we wished to rely on parallel measures across CAPS and FS. However, Principal Component Analyses indicated that in both studies and for both relationships, all three constructs loaded on one factor, strongly supporting the legitimacy of their aggregate as reflecting MRO. The Eigenvalues and percents of variance explained by the one-factor model were as follows, for mothers and fathers: in CAPS, 1.95, 65%; 2.07, 69%; and in FS, 1.95, 65%; 2.25, 75%.

Kim, S., & Kochanska, G. (2012). Child temperament moderates effects of parent-child mutuality on self-regulation: A relationship-based path for emotionally negative infants*. Child Development,* *83*(4), 1275–1289. DOI: 10.1111/j.1467-8624.2012.01778.x

Kochanska, G., & Murray, K. T. (2000). Mother-child mutually responsive orientation and conscience development: From toddler to early school age. *Child Development, 71*(2), 417-431. doi.org/10.1111/1467-8624.00154
