# Supplementary material for: Early relational origins of Theory of Mind: A two‐study replication
Source: J Child Psychol Psychiatry. 2024 Jul 18;66(1):41–52. doi: 10.1111/jcpp.14029 (PMC11652414; doi:10.1111/jcpp.14029)
Supplement: Supplementary file 1 — Table S1. CAPS: Demographic characteristics of the recruited sample at entry (N = 200). Table S2. FS: Demographic characteristics of the recruited sample at entry (N = 102). Appendix S3. The construction of MRO measure [file JCPP-66-41-s001.zip › Table_S2_FS_Demographics_April26.docx]

# Early Relational Origins of Theory of Mind: A Two-Study Replication

Table S2

*FS: Demographic characteristics of the recruited sample at entry (N = 102)*

| Characteristic | *M* or % | *SD* |
| --- | --- | --- |
| Child gender | 50% girls |  |
| Child age at entry (months) | 7.21 | 0.43 |
| Family annual income |  |  |
| Less than $10,000 | 2.0% |  |
| $10,001 – $20,000 | 5.9% |  |
| $20,001 – $30,000 | 8.8% |  |
| $30,001 – $40,000 | 7.8% |  |
| $40,001 – $50,000 | 16.7% |  |
| $50,001 – $60,000 | 8.8% |  |
| $60,001 – $70,000 | 14.7% |  |
| More than $70,001 | 34.3% |  |
| Unknown | 1% |  |

| Mothers | |  | Fathers | |
| --- | --- | --- | --- | --- |
| Characteristic | *M* or % | *SD* | *M* or % | *SD* |
| Age (years) | 30.80 | 5.30 | 32.23 | 6.03 |
| Education |  |  |  |  |
| Did not complete high school | 2.9% |  | 2.9% |  |
| High school | 21.6% |  | 26.5% |  |
| Associate degree | 14.7% |  | 17.6% |  |
| Bachelor’s degree | 39.2% |  | 33.3% |  |
| Advanced degree | 20.6% |  | 19.6% |  |
| Unknown | 1% |  | 0% |  |
| Race^a^ |  |  |  |  |
| Asian | 1.0% |  | 2.0% |  |
| Black or African American | 1.0% |  | 2.9% |  |
| White | 91.2% |  | 84.3% |  |
| Pacific Islander | 1.0% |  | 0% |  |
| Hispanic | 2.9% |  | 7.8% |  |
| More than one race, other | 2.9% |  | 2.0% |  |
| Unknown | 0% |  | 1% |  |

^a^ The race categories listed were used as the official codes at the time of recruitment. “Hispanic” was a race category and not a separate ethnicity dimension.
